# Vertical ex vivo dermoscopy in diagnosing and differentiating skin lesions^؆^

**DOI:** 10.1016/j.abd.2025.501209

**Published:** 2025-10-16

**Authors:** Mirjana Popadić, Dimitrije Brasanac, Danijela Milošev, Ana Ravić Nikolić, Slobodanka Mitrović

**Affiliations:** a Department of Dermatovenerology, Faculty of Medicine, University of Belgrade, University Clinical Centre Belgrade, Serbia; b Institute of Pathology, Faculty of Medicine, University of Belgrade, Serbia; c Department of Pathology, University Clinical Centre Kragujevac, Serbia; d Department of Dermatovenerology, Faculty of Medical Sciences, University Clinical Centre Kragujevac, Serbia; e Department of Pathology, Faculty of Medical Sciences, University Clinical Centre Kragujevac, Serbia

**Keywords:** Dermoscopy, Ex vivo, Vertical view/aspect, Diagnosis, Pigmented skin lesions, Benign skin lesions, Melanocytic nevus, Keratosis seborrheic, Hemangioma, Acanthoma

## Abstract

**Background:**

Publications on vertical *ex vivo* dermoscopy of skin lesions are sporadic and present only as case reports.

**Objectives:**

This study defines, describes, and determines the importance of structures visible by vertical *ex vivo* dermoscopy in the diagnosis of pigmented, benign skin lesions and their distinction between themselves, and in relation to other skin lesions.

**Methods:**

The prospective, descriptive study was conducted in two University centers. Digital images of completely excised skin lesions, fixed in formalin before further processing, were evaluated dermoscopically. For the analysis of horizontal *ex vivo* dermoscopy, pattern analysis was used, while for the vertical section, a description of the visible structures was made.

**Results:**

The research sample consisted of 80 benign pigmented skin lesions obtained from 73 patients. Histopathological diagnosis of the evaluated lesions included 59 nevi, 17 seborrheic keratoses, 3 angiomas and 1 clear cell acanthoma. Seborrheic keratosis had the most variable presentation on vertical *ex vivo* dermoscopy. By analyzing the vertical section of the evaluated skin lesions, the importance of vertical *ex vivo* dermoscopy in the diagnosis and/or differentiation of blue nevus, seborrheic keratosis and angioma from other skin lesions was noted.

**Study limitations:**

Small number of analyzed lesions, and inclusion of Caucasian patients only.

**Conclusions:**

Vertical *ex vivo* dermoscopy can contribute to the distinction between different pigmented benign skin lesions, as well as to their differentiation from other skin lesions. Furthermore, vertical section enables more accurate assessment of additional, descriptive lesional characteristics.

## Introduction

Diagnosis of skin lesions primarily involves a clinical examination (inspection and/or palpation), which has been sufficient in most cases. However, due to global changes (migration of population, changed climatic conditions, appearance of new viruses), many skin lesions are no longer typical and easily recognizable. Application of dermoscopy as a supplementary method enables more accurate diagnosis, by visualizing additional morphological (pigment and/or vascular) criteria.^1^, ^2^, ^3^

Dermoscopy was designed primarily for the diagnosis and differentiation of pigmented skin lesions. However, over time, the indication spectrum for the use of dermoscopy has expanded to include non-pigmented changes, primarily malignant. In the further course, the application of dermoscopy has been extended to inflammatory, parasitic, hair and nail diseases, capillaroscopy, so that today dermoscopy is an indispensable part of the clinical examination.^1^, ^2^, ^3^, ^4^, ^5^

The further development of dermoscopy was aimed at the application of dermoscopy by doctors of other specialties, not only dermatologists. Numerous papers present *ex vivo* dermoscopy, which is a new type of application of standard in vivo dermoscopy on freshly excised tissue or tissue fixed in formalin.^6^, ^7^, ^8^, ^9^ Despite the negative sides of *ex vivo* dermoscopy (poor image quality, loss of vascular structures, significant color changes), published papers show the usefulness of its application as an auxiliary method available to the pathologist (directing the tissue section by indicating the field of interest for the vertical section).^5^, ^8^, ^9^, ^10^, ^11^

A few published papers describe vertical *ex vivo* dermoscopy, which involves the application of dermoscopy on a vertical section of excised, fresh or fixed tissue.^12^ A vertical plane view can be useful to surgeons practicing Mohs micrographic surgery for mapping positive margins.^13^ Due to the possibility of easy sharing of mobile photos of marked positive margins with the surgeon, a possible application is suggested in cases where the Mohs laboratory is not included in the surgical area.^13^

The authors recently published results of vertical ex-vivo dermoscopy analysis of malignant skin lesions, which provides diagnostic information that could be valuable in clinical practice.^14^ Herein is the second part of the investigation, describing the results of vertical ex-vivo dermoscopy in benign pigmented skin lesions.

## Materials and methods

After the approval of the institutional Ethics Committee, a prospective, descriptive study was conducted in the field of vertical *ex vivo* dermoscopy. The research included the analysis of pigmented benign lesions collected during the study.

The experimental part of the research was carried out in two University centers: the service for pathological-anatomical diagnostics, University Clinical Center Kragujevac, Faculty of Medical Sciences in Kragujevac, and the Institute of Pathology of the Faculty of Medicine, University in Belgrade. The research included patients whose lesions were completely surgically excised and sent for histopathological verification in the period from September 1 to December 31, 2019. The study was postponed due to the epidemiological situation caused by the COVID-19 epidemic and continued from January 1 to April 1, 2022.

The criteria for inclusion and exclusion of patients are shown in Table 1. Demographic characteristics of patients and clinical data were collected by reviewing histopathological referrals and outpatient records of patients.

**Table 1:** Criteria for inclusion and exclusion of patients in the study.

Inclusion	Exclusion
• Completely surgically removed skin lesion	• Poor quality of dermoscopic images
• Good quality of dermoscopic photographs	• Descriptive histopathological report
• A clear histopathological diagnosis	• Clinically assessed pigmentation of benign lesions < 50%
• Clinically assessed pigmentation of benign lesions ≥ 50%	

The research was performed on tissue material obtained after surgical removal of skin lesions, fixed in formalin before further processing according to the protocol. First, clinical images of the excised tissue were taken. After that, the contact surface of the dermatoscope was covered with a thin, transparent foil tightened with a rubber band for jars, to prevent cross-contamination.^15^ After the dermoscopic photography of the horizontal aspect of the skin lesion, a vertical section of the tissue was made, which was also photographed according to the same previously described protocol.

A mobile phone camera previously connected to a hand-held dermatoscope (Dermlite 3DLN, California, USA) was used for digital clinical and dermoscopic images. Dermoscopic photographs of vertical tissue sections were taken at different magnifications with a mobile phone camera. All digital photos were electronically transferred and saved to a computer for further evaluation. For dermoscopic analysis of horizontal *ex vivo* dermoscopy, pattern analysis was applied, which determined the existence of dermoscopic structures specific to the type of lesion.^16^ By analyzing *ex vivo* dermoscopic photographs of tissue on a vertical section, a description of the available visible structures was made.

Apart from demographic characteristics of patients (age, gender), independent variables of excised lesions were also evaluated, i.e., localization and diameter of included lesions, and expression of dermoscopic structures on digital photographs of horizontal and vertical aspects of excised skin tissue.

The obtained data were entered into an Excel (Microsoft Office 2016) database for further statistical processing.

### Statistical analysis

A commercial software package (version 22.0, SPSS Inc., Chicago, IL) was used for statistical processing of the obtained results. Histopathological finding was the gold standard of the study. The descriptive statistics methods used in the analysis of the obtained results were:

• Absolute and relative numbers, n (%).
• Measures of central tendency (arithmetic mean – X, median – Med).
• Measures of variability (Standard Deviation ‒ SD, interval of variation).

## Results

### Characteristics of patients and pigmented benign skin lesions

The research sample consisted of 80 benign, pigmented skin lesions obtained from 73 patients, with a predominance of the female gender F = 47 (64.4%), M = 26 (35.6%). The age of the patients ranged from 7 to 83 years (X 44, Med 43.5). The size of the lesions ranged from 1 mm to 25 mm (X7 mm, Med 6 mm). The trunk was the location for 50% (40/80) of the lesions, while 23/80 (28.7%) of the lesions were localized on the extremities, and 17/80 (21.3%) on the head and neck region. Almost all included patients had one lesion (67/73, 91.8%), five patients had 2 lesions (5/73, 6.8%), while only one patient had 3 lesions (1/73, 1.4%).

Histopathological diagnosis of pigmented, benign skin lesions is shown in Table 2.

**Table 2:** Histopathological diagnosis of pigmented benign skin lesions.

Nevus	n=59
Common intradermal	32
Common epidermo-dermal (compound)	19
Dysplastic	6
Reed nevus	1
Blue nevus	1

Seborrheic keratosis	n=17
Acanthotic	7
Irritated	6
Hyperkeratotic	4

Angioma	3
Clear cell acanthoma	1
In total	n=80

### Description of benign pigmented lesions using vertical ex vivo dermoscopy

#### Melanocytic nevus

Among the included lesions, the most common were melanocytic nevi 59/80 (73.75%), and among them, intradermal nevi were the most frequent 32/59 (54.2%).

#### Intradermal nevus

**Fig. 1:**
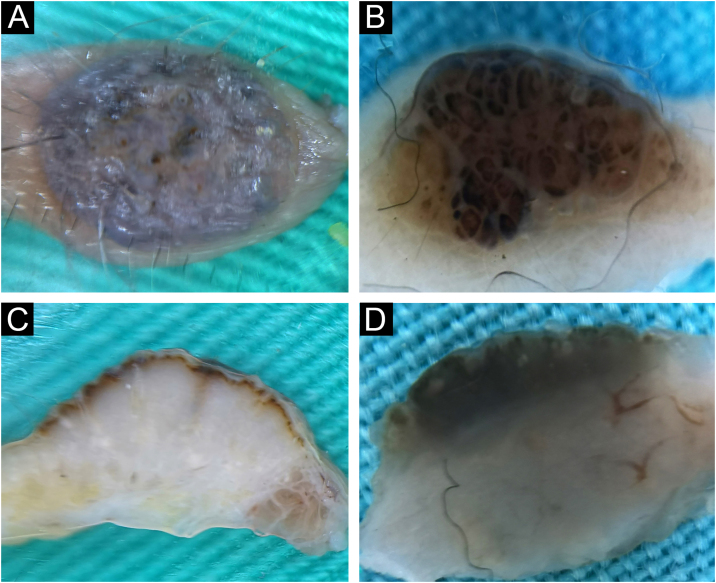
Intradermal nevus. Horizontal *ex vivo* dermoscopy. Presentation as (A) dark blue homogeneous pigmentation. (B) Cobblestone global presentation. Vertical *ex vivo* dermoscopy. (C) Pigmentation in the form of a thin brown line extending above the whitish body with an indistinct lower border. (D) Pigmentation through the entire thickness of the nevus.

Intradermal nevi clinically presented classical features in 12/32 (37.5%), in the form of variably elevated papules, smooth in 11/32 (34.4%), or with papillomatous surface in 9/32 (28.1%). Classic intradermal nevi in the form of soft, hemispherical-shaped, pigmented papules with the presence of a variable number of terminal hairs, on horizontal *ex vivo* dermoscopy, showed brown/blue pigmentation in the homogeneous distribution (Fig. 1A), in a cobblestone pattern (Fig. 1B), or in the form of a network/pseudo-network.

On the vertical section, in more than half of classic intradermal nevi 7/12 (58.3%) pigmentation was presented in the form of a line that followed the rise of the nevus (Fig. 1C). A smaller number of classic intradermal nevi 5/12 (41.7%) on the vertical section showed a variable shade of brown and bluish-brown pigmentation throughout the thickness of the nevus with more developed vascularization (Fig. 1D).

**Fig. 2:**
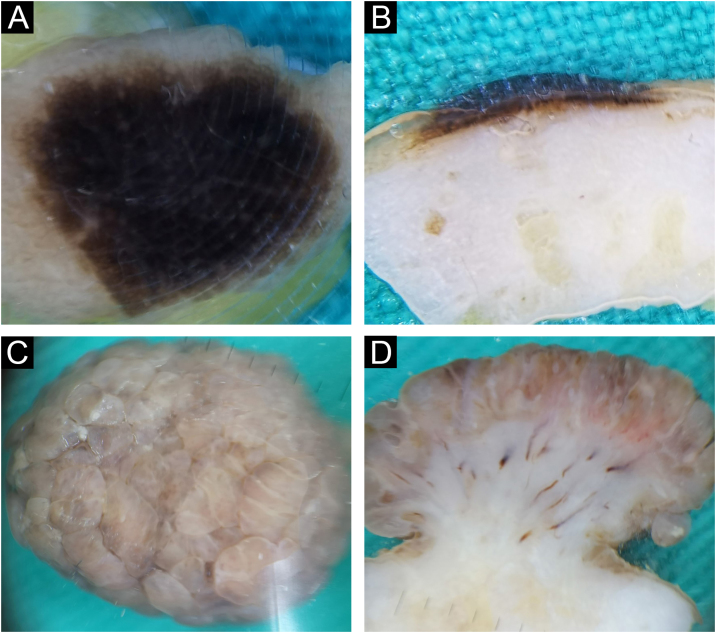
Intradermal nevus. Horizontal *ex vivo* dermoscopy. (A) homogeneous global pattern. (C) cobblestone global pattern. Vertical *ex vivo* dermoscopy. (B) dark pigmentation in the form of a slightly curved line, without clear boundaries of the nevus body. (D) mushroom-shaped tumor, pigmentation of the upper third in the form of a strip of wavy surface with vascularized whitish body.

Eleven intradermal nevi (34.4%) had a clinical presentation in the form of variably elevated pigmented papules. On horizontal *ex vivo* dermoscopy, their presentation was in the form of a homogeneous dark brown pigmentation (Fig. 2A). On the vertical section, these nevi showed pigmentation as continuous lines of variable thickness and length (Fig. 2B).

Papillomatous, intradermal nevi were slightly less frequent 9/32 (28.1%). On horizontal *ex vivo* dermoscopy, a cobblestone global pattern dominated (Fig. 2C). On a vertical section, they were presented in the shape of a mushroom with darker papillae and a whitish base interspersed with short, linear blood vessels (Fig. 2D).

#### Epidermo-dermal (compound) nevus

**Fig. 3:**
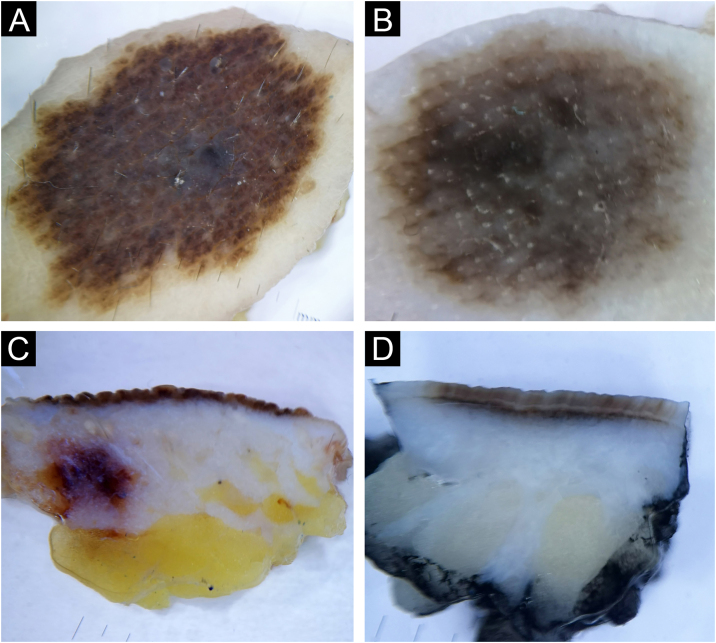
Epidermo-dermal nevus. Horizontal *ex vivo* dermoscopy. (A) globular global pattern. (B) parallel furrow pattern (acral nevus). Vertical *ex vivo* dermoscopy. (C‒D) linear pigmentation of different length, thickness, and color.

There were almost twice as many epidermal-dermal nevi as intradermal nevi, 19/59 (32.2%). Clinically, they were presented in the form of macules and papules with mostly smooth, less often papillomatous surfaces. The global presentation on horizontal *ex vivo* dermoscopy was highly variable ‒ globular, homogenous, reticular, or parallel global pattern (Fig. 3A‒B). However, despite the variable clinical and horizontal dermoscopic presentation, on vertical section, the structure was similar, in the form of a line recolored according to the depth of melanin (Fig. 3C‒D).

#### Dysplastic nevus

**Fig. 4:**
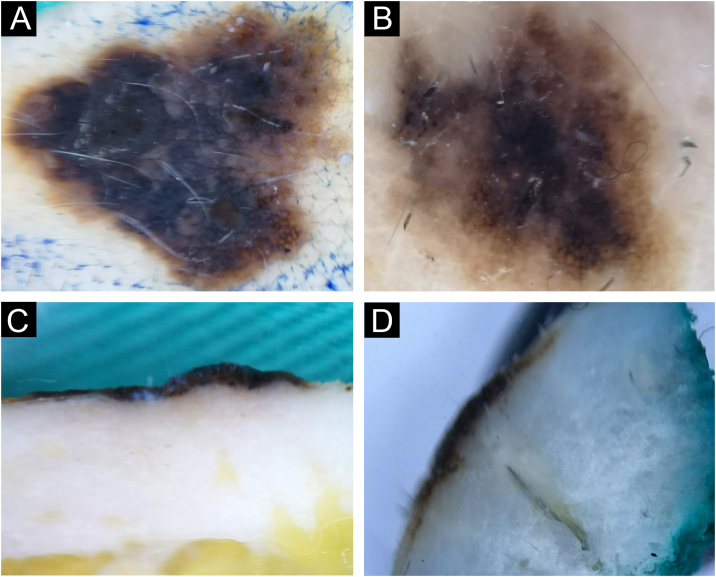
Dysplastic nevus. Horizontal *ex vivo* dermoscopy. (A‒B) Predominance of darkly pigmented homogeneous global pattern with conspicuous presence of one or more dermoscopic features of melanoma. Vertical *ex vivo* dermoscopy. (C‒D) Superficial, thin, linear dark pigmentation.

Histologically confirmed dysplastic nevi were a total of 6/59 (10.2%). Clinically, dysplastic nevi were presented as pigmented macules or papules with positive ABCD criteria. Dysplasticity was also confirmed by horizontal *ex vivo* dermoscopy by the general presence of chaos in the structure and color, with the presence of several criteria for the diagnosis of melanoma (Fig. 4A‒B). Here, as well as with the epidermo-dermal nevus, despite the very different clinical and horizontal dermoscopic presentation, on the vertical section, the dermoscopic presentation was quite similar (Fig. 4C‒D).

#### Blue nevus and Reed nevus

**Fig. 5:**
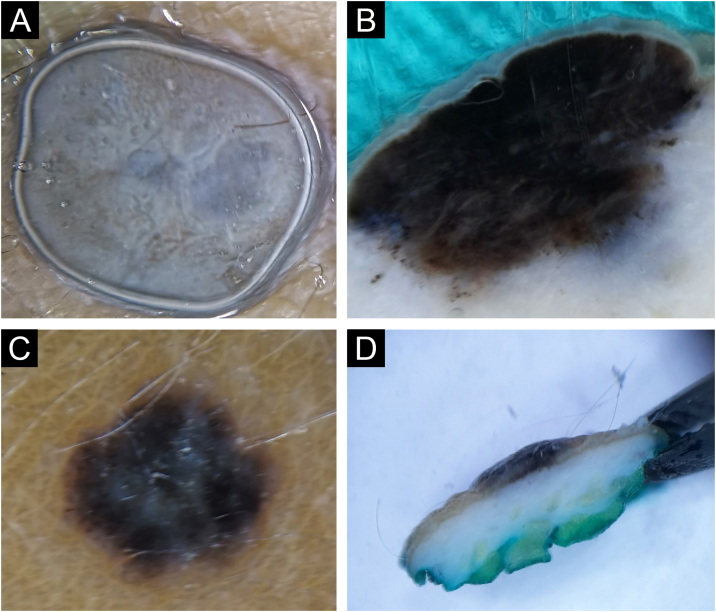
Blue (A‒B) and Reed (C‒D) nevus. Horizontal *ex vivo* dermoscopy. (A) Homogeneous blue global presentation. (C) Homogeneous global pattern, asymmetric shape, irregular edges, and blue-whitish veil in the central part. Vertical *ex vivo* dermoscopy. (B) Visible deep, homogeneous, dark pigmentation with an irregular lower margin. (D) Linear, superficial dark pigmentation.

One blue nevus and one Reed nevus 1/59 (1.7%), were included in the research sample. The clinical and horizontal *ex vivo* presentation of the blue nevus was typical for this nevus (Fig. 5A). On the vertical section, the authors see the true depth of its spread (Fig. 5B).

Reed nevus was clinically presented as a dark pigmented macule, asymmetrical in shape. Horizontal *ex vivo* dermoscopy showed a homogeneous global pattern (Fig. 5C). On a vertical section, it had a similar presentation as a dysplastic nevus (Fig. 5D).

#### Seborrheic keratosis

**Fig. 6:**
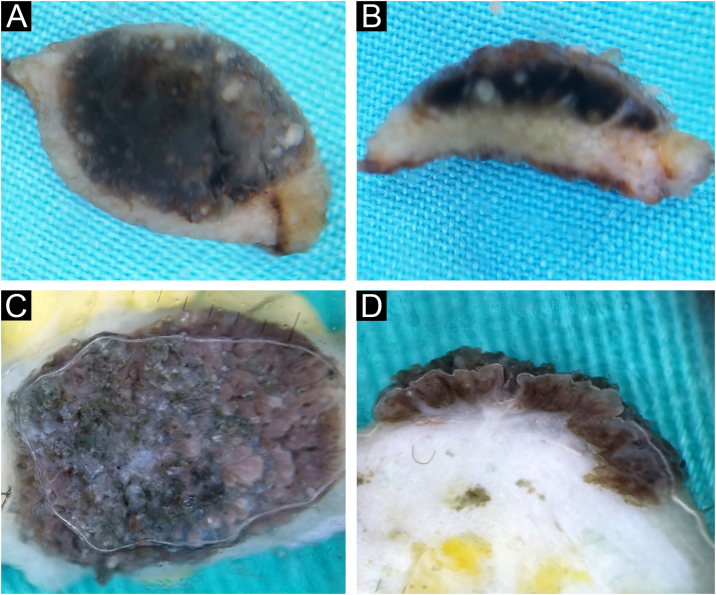
Seborrheic keratosis, acanthotic type (A‒B) and irritated (C‒D). Horizontal *ex vivo* dermoscopy. (A) Milia-like cysts, clear demarcation, homogeneous pigment background. (C) Pronounced hyperkeratosis and papillomatous surface. Vertical *ex vivo* dermoscopy. (B) Thickened epidermis, milia-like cysts, dark pigmentation filling the epidermis. (D) Absence of milia-like cysts, brown, thickened wavy epidermis.

A fifth of the analyzed benign lesions consisted of seborrheic keratoses 17/80 (21.25%), namely acanthotic type 7/17 (41.2%), irritated 6/17 (35.3%), and hyperkeratotic type 4/7 (23.5%). Clinically, acanthotic seborrheic keratoses presented as keratotic, pigmented papules. The majority of seborrheic keratoses of the acanthotic type showed classic characteristics of seborrheic keratosis on both horizontal and vertical *ex vivo* dermoscopy (Fig. 6A‒B).

Irritated seborrheic keratoses did not have a specific clinical presentation. Also, the classical characteristics of seborrheic keratosis were less frequently manifested in both planes of *ex vivo* dermoscopy in the irritated type (Fig. 6C‒D).

Hyperkeratotic seborrheic keratosis was clinically presented as a pigmented, hyperkeratotic papule/plaque. On horizontal *ex vivo* dermoscopy, a global cobblestone pattern was demonstrated, with no visible classic dermoscopic features of seborrheic keratosis. The presentation on the vertical section was more uniform in the form of hyperkeratotic epidermis and unchanged dermis.

#### Angioma

**Fig. 7:**
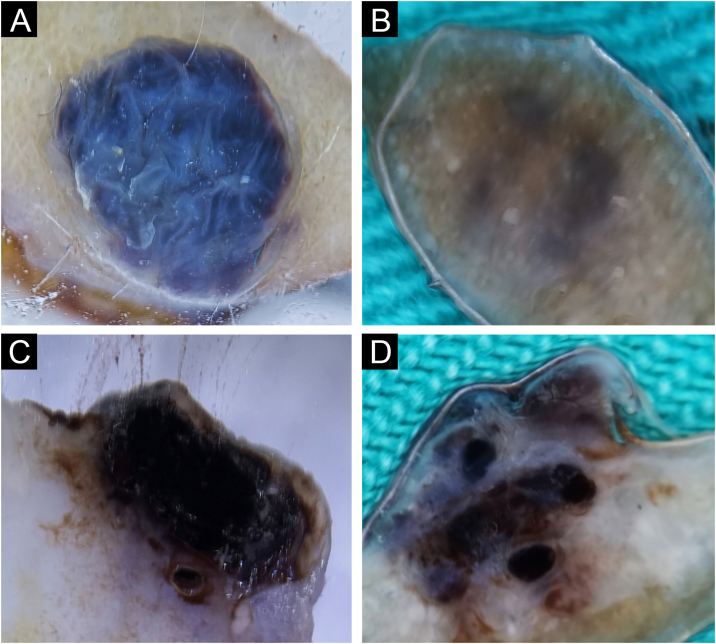
Angioma. Horizontal *ex vivo* dermoscopy. (A) Typical presentation as homogeneous dark blue pigmentation. (B) Atypical presentation as asymmetric, homogeneous light blue pigmentation. Vertical *ex vivo* dermoscopy. (C) Deep homogeneous indigo-black pigmentation, irregular in shape. (D) Typical lacunae and deep homogeneous indigo dark background.

Out of a total of 80 benign lesions, angiomas were 3/80 (3.75%). The clinical presentation was in the form of bluish hemispherical/plane papules. On the horizontal section, the standard blue color of the angioma is visible with the absence of the specific lacunae (Fig. 7A‒B). Darker pigmentation dominates on the vertical section compared to the horizontal plane, showing the true depth of its spread (Fig. 7C‒D).

One out of three angiomas had a nonspecific finding on horizontal *ex vivo* dermoscopy (Fig. 7B), where differentially atypical blue nevus or melanoma cannot be excluded. However, a vertical section reveals structures that clearly indicate the diagnosis of angioma (Fig. 7D).

#### Clear cell acanthoma

Only one clear cell acanthoma was included in this study, 1/80 (1.25%). The clinical presentation was in the form of a bluish papule. Presentation on horizontal and vertical *ex vivo* dermoscopy was nonspecific.

### Diagnosing and differentiating benign pigmented lesions using vertical ex vivo dermoscopy

**Fig. 8:**
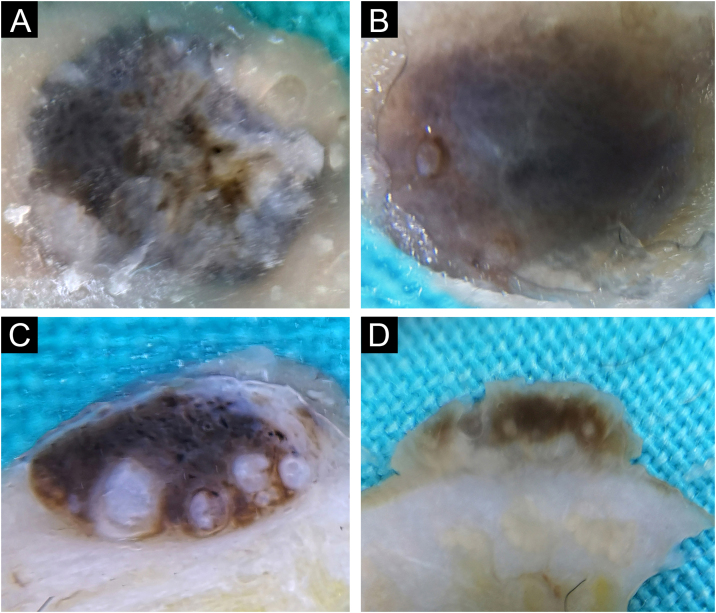
Irritated seborrheic keratosis. Horizontal *ex vivo* dermoscopy. (A) Whitish fields, blue-white veil, asymmetry of color and shape. (B) Non-specific blue-brown pigmentation. Vertical *ex vivo* dermoscopy. (C‒D) Epidermal involvement, striking milia-like cysts, clear regular margins.

During further research, the possibility of diagnosing and differentiating benign pigmented lesions using vertical *ex vivo* dermoscopy was established. In Figs. 7B, and 8A‒B, the authors see pigmented lesions of non-specific findings on horizontal *ex vivo* dermoscopy. However, visible structures on the vertical section clearly indicate the diagnosis of angioma and seborrheic keratosis, respectively (Figs. 7D, and 8C‒D).

**Fig. 9:**
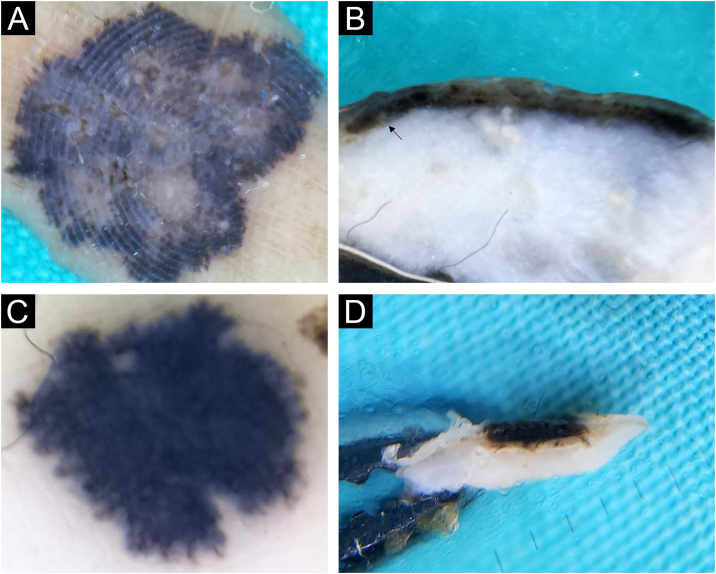
(A‒B) Seborrheic keratosis, acanthotic type. (C‒D) Dysplastic nevus. Horizontal *ex vivo* dermoscopy. (A‒C) Numerous pseudopods along the periphery of the tumor with indigo blue pigmentation interspersed with whitish structures. Vertical *ex vivo* dermoscopy. (B) Lesion confined to the epidermis, pigmentation in the form of a line with a regular lower margin and the presence of one milia-like cyst (arrow). (D) Superficial, dark homogeneous linear pigmentation with tiny islands of pigmentation protruding from the lower margin.

In Fig. 9A‒C, the authors see a seborrheic keratosis and a dysplastic nevus, which on a horizontal section meet the criteria for the diagnosis of melanoma. On the vertical section of both lesions, the authors see a similar presentation, with the presence of small, pigmented islands below the lower margin of the dysplastic nevus, which indicates a deeper than epidermal pigmentation, in contrast to the lower margin of seborrheic keratosis, which clearly limits epidermal pigmentation (Fig. 9B‒D).

**Fig. 10:**
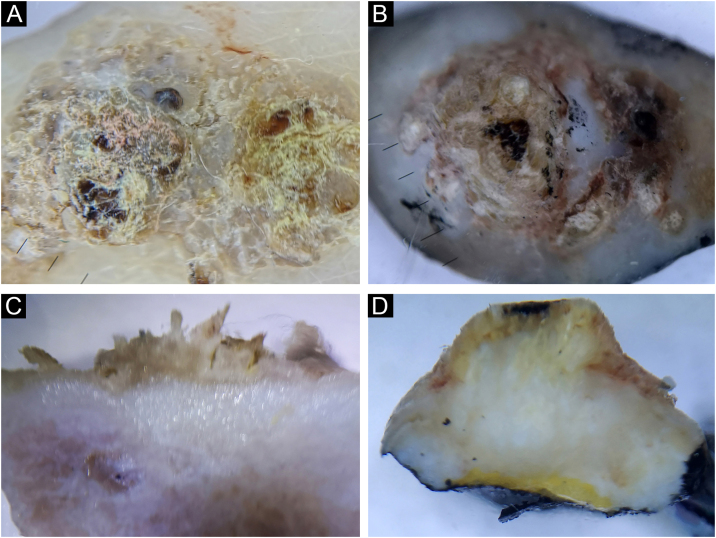
(A‒C) Seborrheic keratosis, hyperkeratotic type. (B‒D) Invasive squamous cell carcinoma. Horizontal *ex vivo* dermoscopy. (A‒B) Visibility of whitish structures, crusts, yellowish scales. Vertical *ex vivo* dermoscopy. (B) Involvement of the epidermis alone, in the form of whitish hyperkeratosis of an irregular surface, with normal dermis. (D) Hyperkeratosis of the epidermis under which there is a whitish tumor body infiltrating the dermis and the subcutaneous fat.

Also, the possibility of differentiation between benign and malignant lesions was investigated using vertical *ex vivo* dermoscopy. Fig. 10A‒B shows a similar, inconclusive finding of both seborrhic keratosis and squamous cell carcinoma on horizontal *ex vivo* dermoscopy. In this case, the vertical section showed the involvement of skin layers and the depth of tumor extension (Fig. 10C‒D).

## Discussion

Despite numerous scientific papers confirming the utility of dermoscopy in daily practice, few papers have actually been published on *ex vivo* horizontal dermoscopy, while publications on *ex vivo* dermoscopy in the vertical plane include only sporadic case reports.^17^, ^18^ Published papers on horizontal *ex vivo* dermoscopy show utility for pathologists,^10^ while for dermatologists and other clinicians involved in dermoscopy, the application of horizontal *ex vivo* dermoscopy does not provide any additional significant diagnostic information compared to in vivo standard dermoscopy. Moreover, a certain amount of diagnostic information is lost by interrupting the vascularization on the tissue section, which is the reason for its non-application in daily clinical practice.^11^

Vertical *ex vivo* dermoscopy has been the least researched, which is why the main goal of this study was to define and describe the structures that can be seen on a vertical section of the tissue and then to determine the possibility of the existence of additional characteristics that could be significant in diagnosis and/or differentiation of pigmented, benign skin lesions.

### Diagnosis and differentiation of benign pigmented skin lesions

#### Melanocytic nevus

Clinical and dermoscopic diagnosis of a nevus is generally easy and does not require *ex vivo* dermoscopy. However, the authors were interested in whether, in rare cases of unclear diagnosis, vertical *ex vivo* dermoscopy can contribute to a more accurate diagnosis and/or differentiation of pigmented benign skin lesions. This study included the analysis of epidermo-dermal, intradermal, dysplastic nevi, and one blue and Reed nevi each.

On the vertical section of the nevus, different forms of pigmentation were seen. Intradermal papillomatous nevi had a unique form of pigmentation, which in the vertical plane presented the shape of a mushroom, made of vascularized whitish body, where the pigmentation covered the entire papillae. In all other evaluated nevi, on the vertical section, two basic forms of pigmentation were seen: superficial pigmentation, the different shape of which (flat, arcuate, hemispherical) follows the elevation of the nevus in the form of a line of variable thickness, length and shade of color and pigmentation throughout the thickness of the nevus.

Pigmentations most often manifested different shades of the natural color of melanin, i.e., brown color. Visible colors on vertical *ex vivo* dermoscopy were correlated with the localization of melanin, i.e., darker shades of brown and black indicated the epidermal localization of melanin, while gray and blue indicated the dermal localization of the pigment. However, the blue nevus showed an exception to this rule when it comes to vertical dermoscopy. The blue color is visible clinically, on standard in vivo, as well as on horizontal *ex vivo* dermoscopy, on a vertical section, is dark brown to black throughout the thickness of the nevus (Fig. 7C-D). Also, apart from the blue nevus, one intradermal nevus showed a deviation from the rules of color and pigment localization on vertical dermoscopy. Visible blue pigmentation in the horizontal plane, on the vertical section, was presented as surface linear brown pigmentation (Fig. 1C).

Further analysis revealed the possibility of differentiating a blue nevus, in cases where clinically and by horizontal dermoscopy the authors see different shades of blue, as was the case with intradermal nevus (Fig. 1A) and Reed nevus (Fig. 5C). Both mentioned nevi, on vertical *ex vivo* dermoscopy, had a different presentation compared to the blue nevus. Superficial, darker, linear pigmentation was seen in the intrademal and Reed nevus (Figs. 1C, and 5D), while in the blue nevus, the spread of dark pigmentation made it possible to see the true depth of its spread, which significantly contributes to its differentiation (Fig. 5B) compared to the other two nevi.

By reviewing the literature, the authors did not find any reports of vertical *ex vivo* dermoscopy of melanocytic nevi, so the authors could not compare the findings. However, the authors came across a report of vertical *ex vivo* dermoscopy of an acral melanoma. Maia et al. showed a vertical *ex vivo* dermoscopy of acral melanoma, where melanin, located within intermediate ridges, was clearly visible, reaffirming the parallel pattern of ridges and the diagnosis of acral melanoma.^12^ Although the present study did not include acral melanoma, but a nevus, the authors compared the findings with the aim of establishing the possibilities of differentiation of the biological nature of acral melanocytic proliferations. In acral regions, determining the location of melanin is of vital importance in differentiating acral malignancies from benign proliferations. In cases of more intense pigmentation, it is more difficult to determine whether the pigment is in a groove or a ridge, so in some cases, it is used to cover the lesions with pen ink, which remains in the grooves after wiping with alcohol, which makes it easier to distinguish ridges from grooves.^19^

Horizontal *ex vivo* dermoscopy of the acral nevus included in the study (Fig. 3B‒D), could not exclude acral melanoma due to the presence of several criteria for melanoma (presence of multiple colors, interruption of the parallel groove pattern in the central part and in places on the periphery, general asymmetry of colors and structures. On vertical *ex vivo* dermoscopy, the authors could clearly see parallel brown, narrow lines within the thickened epidermis, with a wider lighter space between them, indicating furrow pigmentation and not ridge pigmentation as in melanoma, thus allowing their differentiation. Certainly, this finding should be confirmed by further research that would include only plantar melanocytic proliferations.

#### Seborrheic keratosis

Seborrheic keratoses generally do not present a diagnostic problem in daily clinical practice. However, in rare situations, especially when they are pigmented and irritated, seborrheic keratoses require further diagnostics. This study included the analysis of acanthotic, hyperkeratitic, and irritated seborrheic keratoses.

Acanthotic seborrheic keratoses mostly had a classic presentation on horizontal *ex vivo* dermoscopy, with the manifestations that are also seen on standard in vivo dermoscopy (comedo-like openings, milia-like cysts, clear limitation).^4^, ^13^ On the vertical section, the tumor tissue was limited to the epidermis, with the recognition of the same typical characteristics of seborrheic keratosis, but hyperkeratotic and irritated seborrheic keratoses less frequently showed their typical characteristics, both in the horizontal and vertical planes. Of the dermoscopic features of seborrheic keratosis, milia-like cysts were identically presented on vertical sections as well as on horizontal dermoscopies.

Further analysis included the possibility of diagnosis and differentiation of seborrheic keratoses in unclear cases, with variable contribution of vertical *ex vivo* dermoscopy. In the case of inconclusive findings of horizontal dermoscopy in irritated seborrheic keratoses, with a vertical section, the authors encountered characteristics that indicated the diagnosis of seborrheic keratoses (involvement of only the epidermis, clear limitation of the pigmented island, regular shape and edges, presence of typical features). However, in the case of acanthotic seborrheic keratoses, where the differential diagnosis of horizontal *ex vivo* dermoscopy included dysplastic nevus/melanoma, the finding of the vertical section suggested melanocytic lesion as well (dysplastic, intradermal nevus, in situ melanoma) and not seborrheic keratosis (Fig. 9B).

Nevertheless, vertical *ex vivo* dermoscopy in certain cases of hyperkeratotic lesions can contribute to the differentiation of seborrheic keratoses from invasive SCC. In Fig. 10, the authors see a similar presentation of seborrheic keratoses and invasive SCC on horizontal *ex vivo* dermoscopy. However, on a vertical section, epidermal hyperkeratosis without changes in the dermis clearly distinguishes seborrheic keratosis from invasive SCC, in which, in addition to epidermal hyperkeratosis, the authors also see deep involvement of the dermis and subcutis by tumor tissue.

The authors believe that in cases of unclear diagnosis, especially in cases that include skin malignancy in the differential diagnosis, it is worth allocating a little more time for a vertical section and analysis. If the authors find typical characteristics of seborrheic keratosis, vertical *ex vivo* dermoscopy can help us in diagnosis, and if they are absent, the authors can look at the depth of infiltration in order to differentiate between benign and malignant proliferations. Certainly, there is also a scenario where the authors will not receive any significant additional information.

#### Angioma

Typical angiomas are easily diagnosed by clinical examination, while standard dermoscopy increases the certainty of the diagnosis. However, in rare cases, angiomas can be atypical, when they require further diagnostics. Two of the three evaluated angiomas, on horizontal *ex vivo* dermoscopy, showed typical bluish pigmentation interspersed with lighter short lines. Typical angiomas on vertical section presented as homogeneous, deep, dark brown pigmentation instead of the expected blue color visible on horizontal dermoscopy.

Of the dermoscopic characteristics of angioma, lacunae are identically presented on vertical section as well as on horizontal dermoscopy.

When it comes to angiomas, the authors do not recommend vertical *ex vivo* dermoscopy as a routine analysis. However, one angioma in this study did not have the typical dermoscopic finding of an angioma in the horizontal plane, and given the partial bluish pigmentation of an asymmetric shape, the differential diagnosis included an atypical blue nevus, possibly melanoma. Analysis of the vertical plane revealed lacunae, easily visible, typical dermoscopic characteristics of angioma^2^ in the form of dark, oval, clearly limited globules. In this case, vertical *ex vivo* dermoscopy contributed to the diagnosis of angioma and its differentiation from atypical melanocytic lesions.

#### Clear cell acanthoma

This study included only one clear cell acanthoma, which is also rarely diagnosed in daily clinical practice. Due to the variable clinical morphology, it is diagnosed mainly histologically. It is most often presented as a red papule on the lower extremities, while pigmentation is rarely seen.^20^ The dermoscopic image of clear cell acanthoma is dominated by the vascular pattern, where the blood vessels are in the form of dots/globules with a characteristic linear or serpentine arrangement reminiscent of a string of pearls on a pale reddish background.^20^

The finding of horizontal *ex vivo* demoscopy of the analyzed acanthoma was atypical, inconclusive in the form of homogeneous bluish pigmentation that indicated an atypical blue nevus or angioma in the differential diagnosis. On a vertical section, the acanthoma was presented as a slightly arched, brown pigmentation with increased vascularization in the central part of the whitish tumor tissue.

Although dermoscopy in both planes did not contribute to the diagnosis of clear cell acanthoma, in cases of unclear diagnosis, the authors’ recommendation is to perform an additional analysis of the vertical section, as it may contribute to narrowing the differential diagnosis. In this case, the authors can rule out blue nevus and angioma by dermoscopic analysis of the vertical section, which shows deep pigmentation in contrast to the superficial pigmentation seen in clear cell acanthoma. It should be noted that the distribution of punctate blood vessels like a string of pearls is not pathognomonic for clear cell acanthoma, but can be seen, among others, in pigmented seborrheic keratosis. In this case, a vertical examination can allow us to confirm or exclude seborrheic keratosis due to the presence or absence of dermoscopic characteristics of seborrheic keratosis.

As the authors expected, due to the rarity of the literature review, we did not come across a description of vertical *ex vivo* dermoscopy of clear cell acanthoma.

## Conclusions

In conclusion, vertical *ex vivo* dermoscopy provides instant insight into significant descriptive characteristics of skin lesions. Using this technique, the possibility of diagnosis and/or differentiation of blue nevus, seborrheic keratosis, and angioma from other pigmented skin lesions was established, as well as distinguishing hyperkeratotic seborrheic keratosis from invasive squamous cell carcinoma.

It should be emphasized that the resolution of vertical *ex vivo* dermoscopy is not sufficient for evaluating changes at the cellular level or detecting narrow tumor infiltrates.

## Financial support

This work was supported by the Faculty of Medical Sciences, University of Kragujevac, Kragujevac, Serbia (Junior Project No 10/21).

## Authors’ contributions

Mirjana Popadić: Study concept and design; data collection, or analysis and interpretation of data; statistical analysis; writing of the manuscript or critical review of important intellectual content; critical review of the literature.

Dimitrije Brasanac: Data collection, or analysis and interpretation of data; critical review of the literature; final approval of the final version of the manuscript.

Danijela Milošev: Data collection, or analysis and interpretation of data.

Ana Ravić Nikolić: Critical review of the literature; final approval of the final version of the manuscript.

Slobodanka Mitrović: Data collection, or analysis and interpretation of data; critical review of the literature; final approval of the final version of the manuscript.

## Research data availability

The entire dataset supporting the results of this study was published in this article.

## Conflicts of interest

The authors declare no conflicts of interest. The funder had no role in the design of the study; in the collection, analyses, or interpretation of data; in the writing of the manuscript; or in the decision to publish the results.
